# Supplementary material for: Synthetic genetic oscillators demonstrate the functional importance of phenotypic variation in pneumococcal-host interactions
Source: Nat Commun. 2023 Nov 17;14:7454. doi: 10.1038/s41467-023-43241-y (PMC10656556; doi:10.1038/s41467-023-43241-y)
Supplement: Supplementary file 3 — Description of Additional Supplementary Files [file 41467_2023_43241_MOESM3_ESM.pdf]

Description of Additional Supplementary Files

File Name: Supplementary Data S1
Description: Strains and Plasmids used in this study.

File Name: Supplementary Data S2
Description: Oligonucleotides used in this study.

File Name: Supplementary Movie S1
Description: Time-lapse fluorescence microscopy of the CRISPRlator strain (VL3757) grown within a microfluidics device. The CRISPRlator strain and design of the microfluidics device used is show in Fig. 2. Images were taken every 5 min.

File Name: Supplementary Movie S2
Description: Time-lapse fluorescence microscopy of the CAPSUlator strain (VL4315) grown within a microfluidics device. The CAPSUlator strain and design of the microfluidics device used is show in Fig. 2 and Fig. 3. Images were taken every 5 min.
