# Supplementary material for: Synthetic genetic oscillators demonstrate the functional importance of phenotypic variation in pneumococcal-host interactions
Source: Nat Commun. 2023 Nov 17;14:7454. doi: 10.1038/s41467-023-43241-y (PMC10656556; doi:10.1038/s41467-023-43241-y)
Supplement: Supplementary file 8 — Reporting Summary [file 41467_2023_43241_MOESM8_ESM.pdf]

nature portfolio

Corresponding author(s): Jan-Willem Veening

Last updated by author(s): Oct 19, 2023

Reporting Summary

Nature Portfolio wishes to improve the reproducibility of the work that we publish. This form provides structure for consistency and transparency in reporting. For further information on Nature Portfolio policies, see our Editorial Policies and the Editorial Policy Checklist.

Statistics

For all statistical analyses, confirm that the following items are present in the figure legend, table legend, main text, or Methods section.

n/a	Confirmed	
☐	☒	The exact sample size (*n*) for each experimental group/condition, given as a discrete number and unit of measurement
☐	☒	A statement on whether measurements were taken from distinct samples or whether the same sample was measured repeatedly
☐	☒	The statistical test(s) used AND whether they are one- or two-sided<br>*Only common tests should be described solely by name; describe more complex techniques in the Methods section.*
☒	☐	A description of all covariates tested
☒	☐	A description of any assumptions or corrections, such as tests of normality and adjustment for multiple comparisons
☐	☒	A full description of the statistical parameters including central tendency (e.g. means) or other basic estimates (e.g. regression coefficient) AND variation (e.g. standard deviation) or associated estimates of uncertainty (e.g. confidence intervals)
☐	☒	For null hypothesis testing, the test statistic (e.g. *F*, *t*, *r*) with confidence intervals, effect sizes, degrees of freedom and *P* value noted<br>*Give P values as exact values whenever suitable.*
☒	☐	For Bayesian analysis, information on the choice of priors and Markov chain Monte Carlo settings
☒	☐	For hierarchical and complex designs, identification of the appropriate level for tests and full reporting of outcomes
☒	☐	Estimates of effect sizes (e.g. Cohen's *d*, Pearson's *r*), indicating how they were calculated

Our web collection on statistics for biologists contains articles on many of the points above.

Software and code

Policy information about availability of computer code

Data collection	The paper clearly states how data was collected, on which equipment and with which software, when relevant. Data and Code availability are stated in the Method section of the Manuscript.
Data analysis	Data analysis is clearly explained and software packages used noted. Scripts are available on https://github.com/veeninglab/Capsulator and on Zenodo: doi/10.5281/zenodo.10021297

For manuscripts utilizing custom algorithms or software that are central to the research but not yet described in published literature, software must be made available to editors and reviewers. We strongly encourage code deposition in a community repository (e.g. GitHub). See the Nature Portfolio guidelines for submitting code & software for further information.

Data

Policy information about availability of data

All manuscripts must include a data availability statement. This statement should provide the following information, where applicable:

- Accession codes, unique identifiers, or web links for publicly available datasets
- A description of any restrictions on data availability
- For clinical datasets or third party data, please ensure that the statement adheres to our policy

Genome sequence data of the CAPSUlator is available at SRA (SRR24464804, https://www.ncbi.nlm.nih.gov/sra/?term=SRR24464804).

Research involving human participants, their data, or biological material

Policy information about studies with human participants or human data. See also policy information about sex, gender (identity/presentation), and sexual orientation and race, ethnicity and racism.

Reporting on sex and gender	NA
Reporting on race, ethnicity, or other socially relevant groupings	NA
Population characteristics	NA
Recruitment	NA
Ethics oversight	NA

Note that full information on the approval of the study protocol must also be provided in the manuscript.

Field-specific reporting

Please select the one below that is the best fit for your research. If you are not sure, read the appropriate sections before making your selection.

☒ Life sciences ☐ Behavioural & social sciences ☐ Ecological, evolutionary & environmental sciences

For a reference copy of the document with all sections, see nature.com/documents/nr-reporting-summary-flat.pdf

Life sciences study design

All studies must disclose on these points even when the disclosure is negative.

Sample size	Non-parametric statistical tests were used to assess phenotypic differences between different GRNs. Sample sizes were chosen to allow for realistic experimental handling as well as having enough statistical power and reduce the use of animals.
Data exclusions	No data were excluded from the analyses.
Replication	At least 3 biological replicates were performed for each experiment.
Randomization	The experiments were not randomized.
Blinding	The Investigators were not blinded to allocation during experiments and outcome assessment.

Reporting for specific materials, systems and methods

We require information from authors about some types of materials, experimental systems and methods used in many studies. Here, indicate whether each material, system or method listed is relevant to your study. If you are not sure if a list item applies to your research, read the appropriate section before selecting a response.

Materials & experimental systems

n/a	Involved in the study	
☒	☐	Antibodies
☐	☒	Eukaryotic cell lines
☒	☐	Palaeontology and archaeology
☐	☒	Animals and other organisms
☒	☐	Clinical data
☒	☐	Dual use research of concern
☒	☐	Plants

Methods

n/a	Involved in the study	
☒	☐	ChIP-seq
☒	☐	Flow cytometry
☒	☐	MRI-based neuroimaging

Eukaryotic cell lines

Policy information about cell lines and Sex and Gender in Research

Cell line source(s)	Detroit-562, Merck 8601804-1VL

Authentication	Cell line directly grown from Merck, authenticated by microscopy.
Mycoplasma contamination	Not tested
Commonly misidentified lines (See ICLAC register)	No commonly misidentified cell lines were used in the study.

Animals and other research organisms

Policy information about studies involving animals; ARRIVE guidelines recommended for reporting animal research, and Sex and Gender in Research

Laboratory animals	Wild-type C57BL/6J (strain 00664) mice were purchased from The Jackson Laboratory (Bar Harbor, ME), and this mouse colony was bred and maintained in NYU Grossman School of Medicine's conventional mouse facility. Adult mice were fed ad lib the PicoLab Rodent Diet 20, a 20% protein diet formulation, and were given water for consumption. All the animals were kept on a light-cycle of 12 hours on, 12 hours off with a temperature in the animal facility of 70°F (±2°F).
Wild animals	No wild animals were used in the study.
Reporting on sex	Infant pups of both sexes were used for colonization studies. Our previous work has shown that there is no difference in the colonization levels between males and females in our infant mouse model. As such, infants of both sexes were used for this work. At least 3 individual pups were used per infection group per experiment.
Field-collected samples	No field collected samples were used in the study.
Ethics oversight	Animal experiments were performed according to the guidelines laid by National Science Foundation Animal Welfare Act (AWA) and the Public Health Service Policy on the Humane Care and Use of Laboratory Animals. NYU's Grossman School of Medicine's Institutional Animal Care and Use Committee (IACUC) oversees the welfare, well-being, proper care, and use of all animals. They have approved the protocols used in this study: IA16-00538.

Note that full information on the approval of the study protocol must also be provided in the manuscript.
